# Is Forced Migration a Barrier to Treatment Success? Similar HIV Treatment Outcomes Among Refugees and a Surrounding Host Community in Kuala Lumpur, Malaysia

**DOI:** 10.1007/s10461-013-0494-0

**Published:** 2013-06-09

**Authors:** Joshua B. Mendelsohn, Marian Schilperoord, Paul Spiegel, Susheela Balasundaram, Anuradha Radhakrishnan, Christopher K. C. Lee, Natasha Larke, Alison D. Grant, Egbert Sondorp, David A. Ross

**Affiliations:** 1 MRC Tropical Epidemiology Group, Department of Infectious Disease Epidemiology, London School of Hygiene and Tropical Medicine, London, UK; 2 Public Health and HIV Unit, United Nations High Commissioner for Refugees, Geneva, Switzerland; 3 United Nations High Commissioner for Refugees, Kuala Lumpur, Malaysia; 4 Infectious Disease Unit, Department of Medicine, Hospital Sungai Buloh, Sungai Buloh, Selangor Malaysia; 5 Department of Clinical Research, London School of Hygiene and Tropical Medicine, Keppel Street, London, UK; 6 Department of Global Health and Development, London School of Hygiene and Tropical Medicine, London, UK

**Keywords:** Refugees, Forced migration, HIV, Antiretrovirals, Outcomes, Adherence

## Abstract

In response to an absence of studies among refugees and host communities accessing highly active antiretroviral therapy (HAART) in urban settings, our objective was to compare adherence and virological outcomes among clients attending a public clinic in Kuala Lumpur, Malaysia. A cross-sectional survey was conducted among adult clients (≥18 years). Data sources included a structured questionnaire that measured self-reported adherence, a pharmacy-based measure of HAART prescription refills over the previous 24 months, and HIV viral loads. The primary outcome was unsuppressed viral load (≥40 copies/mL). Among a sample of 153 refugees and 148 host community clients, refugees were younger (median age 35 [interquartile range, IQR 31, 39] vs 40 years [IQR 35, 48], *p* < 0.001), more likely to be female (36 vs 21 %, *p* = 0.004), and to have been on HAART for less time (61 [IQR 35, 108] vs 153 weeks [IQR 63, 298]; *p* < 0.001). Among all clients, similar proportions of refugee and host clients were <95 % adherent to pharmacy refills (26 vs 34 %, *p* = 0.15). When restricting to clients on treatment for ≥25 weeks, similar proportions from each group were not virologically suppressed (19 % of refugees vs 16 % of host clients, *p* = 0.54). Refugee status was not independently associated with the outcome (adjusted odds ratio, aOR = 1.28, 95 % CI 0.52, 3.14). Overall, the proportions of refugee and host community clients with unsuppressed viral loads and sub-optimal adherence were similar, supporting the idea that refugees in protracted asylum situations are able to sustain good treatment outcomes and should explicitly be included in the HIV strategic plans of host countries with a view to expanding access in accordance with national guidelines for HAART.

**Electronic supplementary material:**

The online version of this article (doi:10.1007/s10461-013-0494-0) contains supplementary material, which is available to authorized users.

## Resumen

Respondiendo a una ausencia casi total de estudios entre refugiados y las comunidades de acogida y acceso a terapia antirretroviral de gran actividad (TARGA) en zonas urbanas, nuestro objetivo fue comparar la adherencia y resultados virológicos entre los clientes que asisten a una clínica pública en Kuala Lumpur, Malasia. Un estudio transversal se llevó a cabo entre los clientes adultos (≥18 años). Las fuentes de datos incluyen un cuestionario estructurado que midió adherencia auto-reportada, una medida farmacéutica basada en el relleno de medicamentos recetados de TARGA durante 24 meses, y la carga viral del VIH. El resultado principal fue carga viral no suprimida (≥40 copias/mL). Una muestra de 153 refugiados y 148 clientes de la comunidad de acogida fueron reclutados. Los refugiados eran más jóvenes (media de 35 años [rango intercuartil, IQR 31, 39] frente a 40 años [IQR 35, 48], *p* < 0.001), más probabilidades de ser mujer (36 vs 21 %, *p* = 0.004), y haber estado en TARGA durante menos tiempo (61 [IQR 35, 108] vs 153 semanas [IQR 63, 298], *p* < 0.001). Una proporción similar de las personas en tratamiento durante ≥25 semanas de ambos grupos no tuvieron supresión virológica (19 vs 16 %, *p* = 0.54). Las proporciones de cada grupo con <95 % de adherencia a rellenos de recetas de farmacias eran 26 frente a 34 %, *p* = 0.15. La condición de refugiado no se asoció de forma independiente con el resultado (razón de momios ajustado, aOR = 1.28, IC del 95 %: 0.52, 3.14). Las proporciones de refugiados y de clientes de la comunidad de acogida con resultados virológicos no suprimidos y adherencia subóptimas fueron similares, apoyando la idea que los refugiados en situaciones de asilo prolongados son capaces de mantener buenos resultados del tratamiento, y deberían explícitamente incluirse en los planes estratégicos de VIH de los países de acogida con el fin de ampliar el acceso de acuerdo con las directrices nacionales de TARGA.

## Introduction

Global estimates suggest that 8 million people, or 54 % of 14.8 million who are eligible, receive highly active antiretroviral therapy (HAART) [1]. Consistent adherence to HAART is essential for achieving viral suppression and realising the public health benefits of increasing access to treatment. Refugees are persons who have fled across an international border and have a recognised international legal status that should enable them to receive access to medical care on an equivalent basis to host nationals in their countries of asylum [2]. Given potential obstacles such as language barriers, lack of employment and risk of further displacement to other countries [3, 4], there are concerns as to whether refugees who have initiated HAART are sufficiently stable and therefore capable of sustaining optimal adherence and viral suppression. In some instances, governments may be reluctant to provide treatment to refugees [5], citing concerns about stability and the prerogatives of supplying medications to their own citizens. Previous studies among conflict-affected groups reported high levels of adherence and good treatment outcomes, suggesting that barriers may be overcome; however, most of this work was conducted in Sub-Saharan Africa or with refugees based in high-income countries [6]. There are few data available to verify the acceptability of treatment outcomes among refugees in relation to surrounding host communities in Asian settings, where over a third of the world’s 10.6 million refugees were situated as of 2010 [7]. In response, our objective was to study and HIV treatment outcomes among refugee and host community clients accessing HAART from the same clinic in Kuala Lumpur, Malaysia. We hypothesized that refugees would exhibit inferior outcomes when compared with the surrounding host community.

## Method

### Study Setting

Sungai Buloh Hospital, Kuala Lumpur, Malaysia was chosen as the study setting as it met our criteria of an urban, Southeast Asian setting, with sufficient numbers of refugees accessing HIV treatment and care services from a single point of care. At the start of the study (April 2010), 91,985 individuals were registered by the United Nations High Commissioner for Refugees (UNHCR) as refugees or asylum seekers in Malaysia, 315 had an HIV diagnosis, and 171 were on HAART. Over 98 % of refugees on HAART were from Myanmar. By the end of 2009, an HIV-positive refugee remained in Malaysia for an average of 3.7 years; 32 % were resettled to high-income countries after an average of 2.9 years (UNHCR Representation in Malaysia, Pers. Comm). Malaysia has not signed the 1951 Refugee Convention and its 1967 Protocol; however, the Ministry of Health issued a circular in 2006 that permitted refugees to access public health services, including antiretroviral therapy (ART) as part of the national HIV treatment and program. Initially not included in national strategic plans [8], refugees were formally included in the 2011–2015 Strategic Plan [9]. The Malaysian host community, comprised primarily of Malay, Chinese and Tamil groups, were fully subsidised by the national treatment program for first-line HAART (usually stavudine, lamivudine and nevirapine) and virological monitoring; second-line treatments were partially subsidised. For refugees, the national program fully subsidised first-line treatments but more expensive first and second-line drugs (e.g. efavirenz; lopinavir/ritonavir) and virological monitoring were paid for by UNHCR. Refugees did not pay out of pocket for treatment. Only refugees, meaning those who possessed documented approval of their refugee status, received subsidised treatment and support. Asylum-seekers were expedited through the Refugee Status Determination process in order to facilitate timely access to treatment, but did not have access to treatment until refugee status was formally confirmed.

### Study Design

A 15-week (April–July 2010) cross-sectional survey, conducted at the Infectious Diseases Clinic, Sungai Buloh Hospital, aimed to recruit all refugees identified by UNHCR as recipients of HAART and a similar number of host community clients attending the same outpatient clinic. Clients were invited to participate if they were ≥18 years of age, on HAART for ≥30 days and gave informed consent. Refugees had routine access to the clinic one day per week, therefore we sought to recruit host community clients on only one other day per week. Those who met the inclusion criteria were recruited consecutively at the time of their regular clinic appointment and were re-contacted if they agreed but were unable to participate at the time of recruitment. In an attempt to obtain a complete sample, all eligible refugee clients on HAART who met the inclusion criteria but were not seen in the clinic during the study period were contacted by telephone or by a community representative. As attempts were made to recruit all refugees known to be on HAART, the number of eligible refugees determined the upward limit on sample size. Power calculations were initially completed using expected numbers of refugees on HAART and expected proportions virologically suppressed. Given a sample size ratio of 1:1, with 150 clients per group (representing 88 % of eligible refugees) and a level of viral suppression of 70 % in the refugee group, the study had an 80 % chance of detecting a 14 % prevalence difference as statistically significant at the 5 % level. Recruitment of the host community on a 2:1 basis lowered the detectable difference to 12 % (net efficiency gain = 14 %), therefore, the 1:1 strategy was considered sufficient for comparison. To assess the extent to which the host community sample was representative, a sampling frame was constructed from which a randomly selected comparison sample of 150 host clients was selected for the purposes of comparing basic demographic characteristics with the recruited sample.

### Data Sources

The primary outcome was unsuppressed viral load (≥40 copies/mL). Data sources included a structured questionnaire with self-reported adherence measures, a pharmacy-based measure of HAART prescription refills over the previous 24 months and HIV viral loads. The structured questionnaire was translated into Bahasa Malaysia, Tamil, Mandarin, Burmese, and Falam (Chin dialect), then back-translated into English. The original and back-translated English versions were reconciled, then adjusted during pre-testing to enhance validity. Key self-reported adherence measures included a retrospective four-day dose-by-dose recall [10] and a retrospective one-month general recall measured on a visual analogue scale (VAS) [11]. Adherence to pharmacy refill schedule was assessed using a pharmacy-based measure of HAART prescription refills, calculated as the proportion of prescribed refills collected divided by the total required refills for up to 24 months prior to the interview date. A successful refill was determined by dividing the number of tablets claimed into the number of tablets required to avoid a personal stock-out, allowing a 14-day grace period for each collection. For all adherence measures, <95 % of doses taken as prescribed was used to signify “sub-optimal adherence”. Blood samples for HIV viral load measurement were collected using routine phlebotomy procedures and analysed using the COBAS Ampliprep/Taqman platform (Roche Diagnostics Systems, Branchburg, NJ, USA).

### Statistical Methods

Socio-demographic characteristics were compared between host and refugee groups using Mann–Whitney tests, chi-square or Fisher’s exact tests and chi-square tests for trend. Risk factors for unsuppressed viral load were evaluated using unconditional logistic regression; effect estimates were odds ratios (OR) and corresponding 95 % confidence intervals (CI). The order of entry of factors into the model was determined using a three-level, forwards, step-wise modelling approach drawing on social action theory [12] to group factors into levels representing treatment “contexts” such as socio-demographic and displacement factors; “self-change processes” such as knowledge scores and self-efficacy; and “action state” factors consisting of adherence measures. After univariable analyses, a “treatment context model” was fitted by adjusting for treatment context factors with *p* < 0.1 in univariable analyses. A “self-change processes model” was fitted by adjusting each new factor by all retained treatment context factors, then adjusting again for any additional factors with *p* < 0.1. An “action state” model was fitted in a similar fashion but adjustment was restricted to factors from previous levels only so that collinear adherence measures would be excluded. The final regression model was obtained by excluding factors with the highest *p* value, sequentially, until all remaining factors met *p* < 0.05. Covariates of interest retained throughout the modelling process included refugee status, age, and time on HAART. Adherence factors were retained in the final model but were not adjusted for in order to avoid over-adjustment bias [13–15].

### Ethical Approval

Ethical approval was received by the Clinical Research Centre and the Medical Research Ethics committee in Malaysia (Approval 3275) and the London School of Hygiene and Tropical Medicine Research Ethics Committee (Approval 5547).

## Results

### Study Population

We recruited 153 refugees and 148 Malaysian adults reflecting 90 % and 81 % participation rates (eligible clients who were seen or contacted and agreed to participate), respectively. The Malaysian group comprised 6 % of the target population of eligible clients (*N* = 2,870) and was similar on most socio-demographic indicators to a randomly sampled host comparison group (Supplementary Table 1). Almost all (95 %) HIV-positive refugees accessing services from the study clinic were Burmese while the host community group was 61 % Chinese, 25 % Malay, and 15 % Tamil or other ethnic groups. The recruited refugee and host community groups were different on a variety of indicators (Table 1). The refugee group was younger (median age 35 vs 40 years, *p* < 0.001), had a higher proportion of women (36 vs 22 %, *p* = 0.006), a shorter median time on HAART (61 vs 153 weeks, *p* < 0.001), a shorter time since HIV diagnosis (113 vs 315 weeks, *p* < 0.001), and a lower most recent routine CD4 count (278 vs 350 cells/μL, *p* = 0.03). Among refugees, the median time of residence in Malaysia was 3.6 years (IQR 2.0, 6.2) and the median time since having received formal refugee recognition was 1.8 years (IQR 1.0, 2.9).

**Table 1:** Baseline socio-demographic and treatment factors among host community (*n*_1_ = 148) and refugee (*n*_2_ = 153) clients

Factor	Host	Refugee^a^	*p* value
Female ∑, *n* (%)	33/148 (22)	55/153 (36)	0.006^b^
Age, median years (IQR)	40 (35, 48)	35 (31, 39)	<0.001^c^
Unemployed, *n* (%)	50/148 (34)	91/152 (60)	<0.001^d^
Educational status, *n* (%)			
None	3/148 (2)	8/153 (5)	<0.001^b^
Any primary	16/148 (11)	60/153 (39)	
Any secondary or above	129/148 (87)	85/153 (56)	
Marital status, *n* (%)			
Single	90/148 (61)	61/153 (40)	<0.001^b^
Married	58/148 (39)	92/153 (60)	
Nationality			
Malaysian	148/148 (100)	0/151 (0)	<0.001^b^
Burmese	0/148 (0)	146/151 (97)	
Other	0/148 (0)	5/151 (3)	
Current defaulters, *n* (%)^e^	16/148 (11)	10/153 (7)	0.19^d^
Viral load, copies/mL (%)			
Suppressed <40	112/144 (78)	112/152 (74)	0.41^d^
Not suppressed ≥40	32/144 (22)	40/152 (26)	
Most recent routine CD4, median cells/μL (IQR)^f^	350 (202, 486)	278 (182, 423)	0.03^c^
Time on HAART, median weeks (IQR)^g^	153 (63, 298)	61 (35, 108)	<0.001^c^
Time since HIV diagnosis, median weeks (IQR)^h^	315 (152, 571)	113 (66, 170)	<0.001^c^
Time since entry to host country, median weeks (IQR)	NA	186 (105, 324)	NA
Time since refugee status approval, median weeks (IQR)^i^	NA	91 (54, 149)	NA

∑ Two Malaysian transgender clients were included as females

^a^Three refugees were traced to the inpatient and TB wards and were retained in analyses (2/3 had supressed viral loads)

^b^Chi-square test

^c^Mann–Whitney test

^d^Fisher’s exact test

^e^1 to 5 consecutive months without pharmacy refill

^f^ *n*_1_ = 140, *n*_2_ = 141

^g^ *n*_1_ = 147, *n*_2_ = 150

^h^ *n*_1_ = 146, *n*_2_ = 153

^i^ *n*_2_ = 152

### Virological and Adherence Outcomes

Viral load results indicated that 24 % (72/296) of clients had not achieved viral suppression (≥40 copies/mL). There was no difference between the proportions of refugees and host community clients who had not achieved viral suppression overall, or when restricting analyses to clients on treatment for ≥25 weeks (19 vs 16 %, *p* = 0.54; Table 2). Among all surveyed clients, both groups performed similarly on key measures of self-reported adherence (Table 3). The four-day recall showed that low proportions of both groups self-reported sub-optimal adherence (8 vs 4 %, *p* = 0.20), whereas the proportions who self-reported sub-optimal adherence on the one-month VAS were higher (28 vs 30 %, *p* = 0.79). The pharmacy refill results were also higher but similar in both groups (26 vs 34 %, *p* = 0.15). Within each group, there was evidence for ordered trends between self-reported measures of adherence and proportions not virologically suppressed among clients on treatment for ≥25 weeks. On the pharmacy refill measure, there was strong evidence for this trend among refugees, but this did not hold for the host community (see Supplementary Table 2).

**Table 2:** Comparison of virological outcomes in host community and refugee clients

Time on HAART (weeks)	Group	≥40 copies/mL, *n* (%)	Total, *n* (%)	*p* value^a^
All^b^	Host	32 (22)	144 (100)	0.41
	Refugee	40 (26)	152 (100)	
<25	Host	12 (67)	18 (100)	1.00
	Refugee	17 (59)	29 (100)	
≥25	Host	20 (16)	125 (100)	0.54
	Refugee	23 (19)	121 (100)	

^a^Chi-square test

^b^5 % (7/147) of client’s with a previous viral load <40 copies/mL tested in the range of 40 to 499 copies/mL. Among clients displaying this low-level viraemia, no differences were observed between the groups (Fisher’s exact test, *p* < 1.00)

**Table 3:** Proportions of refugee and host community clients adhering to HAART by four-day self-reports, one-month self-reports and pharmacy refills

Adherence measure	Host, *n* (%)	Refugee, *n* (%)	*p* value^a^
Dose-by-dose self-report (four days)	(*n* = 148)	(*n* = 153)	0.20
0+	6 (4)	11 (7)	
80+	0 (0)	1 (1)	
95+	142 (96)	141 (92)	
Visual analogue scale self-report (one month)	(*n* = 148)	(*n* = 153)	0.79
0+	11 (7)	11 (7)	
80+	33 (22)	32 (21)	
95+	104 (70)	110 (72)	
Pharmacy claim adherence (24 months)^b^	(*n* = 143)	(*n* = 136)	0.15
0+	14 (10)	9 (7)	
80+	34 (24)	26 (19)	
95+	95 (66)	101 (74)	

^a^Chi-square test for trend (Cochran–Armitage test)

^b^Since started on HAART to a maximum of 24 months, retrospectively

### Risk Factors for Unsuppressed Virological Outcomes

Unsuppressed viral load was defined as ≥40 copies/mL. In initial analyses of contextual factors (Table 4), 17 % of clients on HAART for ≥1 year were not suppressed. Among those on treatment for ≥25 weeks, 15 % of those on HAART for <1 year were not suppressed. There was no significant relationship between increasing time on treatment (over 1 year) and virological outcomes (aOR = 1.17, 95 % CI 0.69, 1.96; *p* = 0.56).

**Table 4:** Association of contextual factors with unsuppressed viral load among refugees and local host community on HAART for ≥25 weeks in Kuala Lumpur, Malaysia (*N* = 222)

Factor	Prevalence ≥40 copies/mL, *n*/*N* (%)^a^	*p* value, crude odds ratio (95 % CI)	*p* value, adjusted odds ratio (95 % CI)^b^
Age group (years)^c^		*p* = 0.69	*p* = 0.68
18−	5/25 (20)	1	1
30−	18/114 (16)	0.90 (0.52, 1.55)	1.15 (0.60, 2.20)
40+	13/83 (16)		
Refugee status		*p* = 0.19	*p* = 0.60
Host	15/114 (13)	1	1
Refugee	21/108 (19)	1.59 (0.77, 3.28)	1.28 (0.52, 3.14)
Time on HAART (years)^c^		*p* = 0.79	*p* = 0.56
0−	7/46 (15)	1	1
1−	9/57 (16)	1.06 (0.68, 1.67)	1.17 (0.69, 1.96)
2+	20/119 (17)		
Sex		*p* = 0.04	*p* = 0.05
Male	30/155 (19)	1	1
Female/transgender	6/67 (9)	0.41 (0.16, 1.04)	0.39 (0.14, 1.05)
Time from diagnosis to start (weeks)^c^		*p* = 0.07	*p* = 0.04
0−	19/98 (19)	1	1
25−	8/30 (27)	0.69 (0.47, 1.03)	0.64 (0.41, 0.99)
50+	9/94 (10)		
HAART regimen, dosing		*p* = 0.32	*p* = 0.13
EFV-based	21/140 (15)	1	1
NVP-based	12/74 (16)	1.10 (0.51, 2.38)	1.03 (0.44, 2.43)
Other	3/8 (38)	3.40 (0.76, 15.31)	6.00 (1.14, 31.74)
Current employment		*p* = 0.23	*p* = 0.21
No	13/101 (13)	1	1
Yes	23/121 (19)	1.59 (0.76, 3.32)	1.70 (0.74, 3.95)
Mother tongue		*p* = 0.19	*p* = 0.26
Bahasa Malaysia (Malay)	5/39 (13)	1	1
Tamil	5/26 (19)	1.62 (0.42, 6.27)	1.56 (0.36, 6.73)
Chinese dialects	3/46 (7)	0.47 (0.11, 2.13)	0.47 (0.09, 2.32)
Chin dialects	13/54 (24)	2.16 (0.70, 6.66)	6.21 (0.57, 67.53)
Burmese	3/24 (13)	0.97 (0.21, 4.49)	2.52 (0.17, 38.58)
Other	7/33 (21)	1.83 (0.52, 6.43)	3.20 (0.30, 34.63)
Household size^c^		*p* = 0.73	*p* = 0.97
1−	9/56 (15)	1	1
3−	17/112 (15)	1.09 (0.66, 1.82)	1.01 (0.59, 1.73)
7+	10/54 (19)		
No. dependent minors in household		*p* = 0.59	*p* = 0.98
0	23/133 (17)	1	1
1+	13/89 (15)	0.82 (0.39, 1.72)	1.01 (0.44, 2.33)
Temporary migration (≥1 continuous month in past year)		*p* < 0.001	*p* = 0.002
No	23/187 (12)	1	1
Yes	13/35 (37)	4.21 (1.87, 9.50)	4.12 (1.70, 9.99)
Pathway to diagnosis		*p* = 0.50	*p* = 0.65
Voluntary test	7/43 (16)	1	1
Mandatory test	8/40 (20)	1.29 (0.42, 3.94)	2.01 (0.56, 7.18)
Illness/hospitalisation	16/88 (18)	1.14 (0.43, 3.03)	1.00 (0.34, 2.93)
Other	5/51 (10)	0.56 (0.16, 1.91)	1.07 (0.27, 4.25)
Average time to clinic (hours)		*p* = 0.01	*p* = 0.02
0−	6/74 (8)	1	1
1+	30/148 (20)	2.88 (1.14, 7.27)	3.05 (1.09, 8.49)
Regimen switch, ever		*p* = 0.20	*p* = 0.07
No	16/120 (13)	1	1
Yes	20/102 (20)	1.59 (0.77, 3.25)	2.14 (0.94, 4.85)
Unable to refill prescription, past three months		*p* = 0.41	*p* = 0.44
No	35/210 (17)	1	1
Yes	1/12 (8)	0.45 (0.06, 3.64)	0.45 (0.05, 4.08)
Any symptom or side-effect, past four weeks		*p* = 0.23	*p* = 0.41
No	6/54 (11)	1	1
Yes	30/168 (18)	1.74 (0.68, 4.44)	1.51 (0.55, 4.19)
Food security^d^		*p* = 0.17	*p* = 0.23
Secure	10/84 (12)	1	1
Insecure	26/138 (19)	1.72 (0.78, 3.77)	1.83 (0.67, 5.00)
Satisfaction with primary health care provider, mean score^e^	Mean = 4.21; SD = 0.70	*p* = 0.85; 0.95 (0.57, 1.59)	*p* = 0.64; 0.88 (0.51, 1.51)

Note: 32 clients with incomplete data were excluded (five missing viral loads; 13 missing pharmacy claim records). Clients with missing data were not significantly different (*p* > 0.05) from those retained for analyses on age, sex, refugee status, and time on HAART

^a^Unless otherwise noted

^b^Adjusted for age group, sex, refugee status, travel in past year, time to clinic, time on HAART, and time from HIV diagnosis to HAART start

^c^Factor modelled as a linear effect (common odds ratios presented)

^d^Item constructed from three questions, each measured on a three-point Likert scale. An endorsement of “some of the time” or “all of the time” on any of the three questions was scored as “insecure”

^e^Item constructed from two questions, each measured on a five-point Likert scale; ascending score was consistent with greater satisfaction

There was no evidence for associations between self-change process factors and the outcome (Table 5). Among exposures in the action state level (Table 6), there was a protective effect of adherence to pharmacy refill schedule (aOR = 0.47, 95 % CI 0.27, 0.83; *p* = 0.009) and a harmful effect of having reported any treatment interruption in the past month (aOR = 2.77, 95 % CI 0.91, 8.43; *p* = 0.08), adjusting for age group, time on HAART, refugee status, sex, temporary travel in past year, time to clinic, time from diagnosis to HAART start and previous regimen switch.

**Table 5:** Association of self-change factors with unsuppressed viral load among refugees and local host community on HAART for ≥25 weeks in Kuala Lumpur, Malaysia (*N* = 222)

Factor	Prevalence ≥40 copies/mL, *n*/*N* (%)	*p* value, crude odds ratio (95 % CI)	*p* value, adjusted odds ratio (95 % CI)^a^
Adherence self-efficacy (self-rated ability to take medications as prescribed over previous month)^b^		*p* = 0.37	*p* = 0.95
Excellent	16/99 (16)	1	1
Good/very good	14/105 (13)	1.30 (0.74, 2.26)	1.02 (0.56, 1.86)
Very poor/poor/fair	6/18 (33)		
Serostatus disclosure to partner		*p* = 0.67	*p* = 0.77
No	4/22 (18)	1	1
Yes	17/120 (14)	0.74 (0.22, 2.46)	1.11 (0.29, 4.23)
No partner	15/80 (19)	1.04 (0.31, 3.52)	1.45 (0.38, 5.53)
Serostatus disclosure to family/friends		*p* = 0.23	*p* = 0.49
No	10/81 (12)	1	1
Yes	26/141 (18)	1.61 (0.73, 3.53)	1.37 (0.56, 3.34)
Alcohol use, past month		*p* = 0.29	*p* = 0.69
Never	24/164 (15)	1	1
One or more times	12/58 (21)	1.52 (0.71, 3.28)	0.83 (0.33, 2.06)
Use of illegal/harmful substances, past six months		*p* = 0.23	*p* = 0.83
No	32/208 (15)	1	1
Yes	4/14 (29)	2.20 (0.65, 7.45)	1.18 (0.27, 5.31)
Use of traditional medicines, past six months		*p* = 0.46	*p* = 0.75
No	29/188 (15)	1	1
Yes	7/34 (21)	1.48 (0.57, 3.57)	1.31 (0.47, 3.70)
No. of reported barriers to adherence^b^		*p* = 0.46	*p* = 0.89
0	13/82 (16)	1	1
1+	8/67 (12)	1.13 (0.82, 1.56)	1.03 (0.71, 1.49)
3+	8/36 (22)		
5+	7/37 (19)		
Knowledge of HIV and AIDS (% correct of four questions)		*p* = 0.15	*p* = 0.23
0+	1/18 (6)	1	1
50+	35/204 (17)	3.52 (0.45, 27.33)	3.21 (0.37, 28.05)

Note: 32 clients with incomplete data were excluded (five missing viral loads; 13 missing pharmacy claim records). Clients with missing data were not significantly different (*p* > 0.05) from those retained for analyses on age, sex, refugee status, and time on HAART

^a^Adjusted for age group, sex, refugee status, travel in past year, time to clinic, time on HAART, time from HIV diagnosis to HAART start, and previous regimen switch

^b^Factor modelled as a linear effect (common odds ratios presented)

**Table 6:** Association of action state (adherence) factors with unsuppressed viral load among refugees and local host community on HAART for ≥25 weeks in Kuala Lumpur, Malaysia (*N* = 222)

Factor	Prevalence ≥40 copies/mL, *n*/*N* (%)	*p* value, crude odds ratio (95 % CI)	*p* value, adjusted odds ratio (95 % CI)^a^
Adherence to medication schedule, self-reported		*p* = 0.44	*p* = 0.81
Never, sometimes, half of the time, most of the time	12/62 (19)	1	1
All of the time	24/160 (15)	0.74 (0.34, 1.58)	0.90 (0.39, 2.08)
Adherence, visual analogue scale self-report, past month (%)^b^		*p* = 0.01	*p* = 0.17
0−	5/13 (39)	1	1
80−	10/46 (22)	0.50 (0.29, 0.86)	0.65 (0.35, 1.19)
95+	21/163 (13)		
Adherence, dose-by-dose self-report, past four days (%)		*p* = 0.04	*p* = 0.30
0−	4/9 (44)	1	1
95+	32/213 (15)	0.22 (0.06, 0.87)	0.32 (0.06, 1.76)
Adherence, pharmacy refill schedule, HAART start or 24 months^b^		*p* = 0.002	*p* = 0.009
0−	8/22 (36)	1	1
80−	12/53 (23)	0.45 (0.28, 0.73)	0.47 (0.27, 0.83)
95+	16/147 (11)		
Treatment interruptions of ≥1 day, self-report, past month		*p* = 0.003	*p* = 0.08
None	27/200 (14)	1	1
Any	9/22 (41)	4.44 (1.73, 11.38)	2.77 (0.91, 8.43)
Unintentional underdosing		*p* = 0.32	*p* = 0.30
No	27/180 (15)	1	1
Yes	9/42 (21)	1.55 (0.67, 3.59)	1.66 (0.65, 4.24)

Note: 32 clients with incomplete data were excluded (five missing viral loads; 13 missing pharmacy claim records). Clients with missing data were not significantly different (*p* > 0.05) from those retained for analyses on age, sex, refugee status, and time on HAART

^a^Adjusted for age group, sex, refugee status, travel in past year, time to clinic, time on HAART, time from diagnosis to HAART start, and previous regimen switch

^b^Factor modelled as a linear effect (single common odds ratio presented)

The final multivariable model (Table 7) identified female sex (aOR = 0.39, 95 % CI 0.14, 1.05; *p* = 0.05), increasing time between diagnosis and treatment start (aOR = 0.64, 95 % CI 0.41, 0.99; *p* = 0.04) and adherence to pharmacy claim schedule (aOR = 0.47, 95 % CI 0.27, 0.81; *p* = 0.007) as protective, while temporary migration of ≥1 month in the past year (aOR = 4.12, 95 % CI 1.70, 9.99; *p* = 0.002) and average travel time to clinic of ≥1 hour (aOR = 3.05, 95 % CI 1.09, 8.49; *p* = 0.02) increased the odds of having an unsuppressed viral load. There was no evidence for an association between refugee status and unsuppressed viral load (aOR = 1.28, 95 % CI 0.52, 3.14; *p* = 0.60) adjusting for age group, refugee status, time on HAART, sex, temporary migration in the past year, average time to clinic, and time from HIV diagnosis to HAART start.

**Table 7:** Final multivariate model for factors associated with unsuppressed viral load among refugees and a local host community on HAART for ≥25 weeks in Kuala Lumpur, Malaysia

Factor	*p* value, crude odds ratio (95 % CI)	*p* value, adjusted odds ratio (95 % CI)^a^
Age group (years)^b^	*p* = 0.69	*p* = 0.68
18−	1	1
30−	0.90 (0.52, 1.55)	1.15 (0.60, 2.20)
40+		
Refugee status	*p* = 0.19	*p* = 0.60
Host	1	1
Refugee	1.59 (0.77, 3.28)	1.28 (0.52, 3.14)
Sex	*p* = 0.04	*p* = 0.05
Male	1	1
Female	0.41 (0.16, 1.04)	0.39 (0.14, 1.05)
Time on HAART (years)^b^	*p* = 0.79	*p* = 0.53
0−	1	1
1−	1.06 (0.68, 1.67)	1.17 (0.69, 1.96)
2+		
Time from diagnosis to start (weeks)^b^	*p* = 0.03	*p* = 0.04
0−	1	1
25−	0.61 (0.39, 0.95)	0.64 (0.41, 0.99)
50+		
Temporary migration (≥1 continuous month in past year)	*p* < 0.001	*p* = 0.002
No	1	1
Yes	4.21 (1.87, 9.50)	4.12 (1.70, 9.99)
Average time to clinic (hours)	*p* = 0.01	*p* = 0.02
0−	1	1
1+	2.88 (1.14, 7.27)	3.05 (1.09, 8.49)
Adherence, pharmacy refill schedule, HAART start or 24 months^b,c^	*p* = 0.002	*p* = 0.007
0−	1	1
80−	0.45 (0.28, 0.73)	0.47 (0.27, 0.81)
95+		

Note: 32 clients with incomplete data were excluded (five missing viral loads; 13 missing pharmacy claim records). Clients with missing data were not significantly different (*p* > 0.05) from those retained for analyses on age, sex, refugee status, and time on HAART

^a^Confounders pre-selected for retention in the final model included age group, refugee status and time on HAART. After the first model run, “previous regimen switch” did not meet *p* < 0.05 and was excluded

^b^Factor modelled as a linear effect (single common odds ratio presented)

^c^Factor not included in the final model process due to presumptive role as mediator; other final model factors were not adjusted for these designated factors

## Discussion

In this study, the first we are aware of that investigated adherence and treatment outcomes by comparing refugees with a host community in an asylum setting, a minority of both refugee (19 %) and host community clients (16 %) on HAART for ≥25 weeks did not achieve viral suppression. Only minor differences were found on self-reported and pharmacy-based adherence measures. Adherence and virological outcomes were comparable to results from other Asian HIV clinics. In a multicentre prospective cohort carried out in 17 Asian settings, Oyomopito and colleagues found that 17 % were not virologically suppressed after 12 months on HAART [16]. We are aware of only one other report of virological outcomes among forcibly displaced or conflict-affected groups situated in low and middle-income settings [17]. In a South African study, 24 % of “foreigners”, many of whom had emigrated from Zimbabwe but who were not explicitly identified as refugees, exhibited a study-specific measure of viral failure that included individuals with a viral load of ≥1000 copies/mL. Previous adherence data collected among other groups in low and middle-income settings have shown results that are consistent with other stable cohorts. In conflict-affected northern Uganda, Kiboneka and colleagues [18] found adherence levels of <95 % in 8 % of internally-displaced persons (IDP), as measured by a composite adherence score. In a Ugandan cross-sectional study of IDPs, mean self-reported adherence was 99.5 % [19]. In the western Equatorial province of Sudan, 12 % of refugees and IDPs on HAART for ≥6 months self-reported <95 % adherence [20]. During active conflict in the Democratic Republic of the Congo, sub-optimal adherence (measured by pill counts) was found in only 1 % of clients while CD4 gain at six months was similar to other stable cohorts [21].

Given the potential for cross-border displacement to increase the vulnerability of refugees to inferior outcomes, it was reassuring that a high proportion of refugees were virologically suppressed in the present study. In multivariable analyses, no independent association was found between refugee status and unsuppressed viral load after adjusting for age, sex, time on HAART, time from diagnosis to HAART start, temporary migration in the past year and time to clinic. Consistent with evidence from a Canadian setting showing an adverse impact of temporary migration [22], travel outside of current residence for ≥1 month in the past year (reported by 18 % of refugees and 14 % of Malaysians) led to a fourfold increase in the odds of unsuppressed viral load, a possible consequence of difficulties locating or refilling medications when personal stocks were depleted in the absence of contingency plans while away. Consistent with other settings, longer travel times to clinic (≥1 h) were linked to an increase in the odds of unsuppressed viral load [23–25]. By contrast, many of the obstacles thought to adversely affect treatment outcomes among refugees such as language barriers, unemployment and instability were either not associated with the outcome or were not unique to refugees. Specifically, there was no evidence for an adverse effect of employment status or language group. Language barriers may have been overcome by the effective use of interpreters and support counsellors recruited directly from refugee communities. We did not study onwards displacement to other countries directly; however, the average length of stay for an HIV-positive refugee (3.7 years) was generally indicative of stability. The finding that temporary migration (for ≥1 continuous month in the past year) was a risk factor after adjusting for refugee status suggested that this was common to the full study group. Longer times between diagnosis and HAART start were protective, even though starting HAART at a higher CD4 counts is known to reduce mortality [26]. Longer lead-in times to routine treatment may have facilitated treatment readiness while the negative impact of delaying treatment may have been confounded by delays between seroconversion and diagnosis. Specifically, clients may have started HAART during acute illness when they were motivated to get well by adhering to treatment.

The finding that women were more likely to have achieved viral suppression could have been due to gender differences in proportions disclosing their status to partners (49 % of males vs 66 % of females, *p* = 0.05) and in proportions with children (40 % of males vs 61 % of females, *p* = 0.004). Non-disclosure of HIV status has previously been shown to affect adherence [27], while having children may provide earlier pathways to care through antenatal screening [28]. The better outcomes observed among women were consistent with results from a Chinese study [29] and a South African study that showed a tendency for men to present for treatment later and with more advanced disease [30].

Sub-optimal pharmacy refill adherence was strongly associated with lack of viral suppression, supporting the usefulness of this measure for routine monitoring especially where viral load measurement is unavailable [31, 32]. The slightly higher proportion of Malaysians not adhering optimally to the pharmacy claim schedule may have been an artefact of a system that facilitated occasional or supplementary medication collection from external pharmacies (refugees did not have similar opportunities). One-sixth of host community clients reported collecting drugs in this manner within the assessed pharmacy refill period. Multiple routine adherence indicators could help to facilitate accurate monitoring of adherence patterns over time [33].

Caution must be used when generalising these findings to other refugee populations given that only one setting was studied and HAART delivery systems are so often setting-specific. The HIV-positive caseload among refugees was considerably higher in Malaysia in comparison to other major programs in the region (ten cases each in Bangkok and New Delhi). Moreover, there are differences between refugee settings (e.g. urban, rural, dispersed, and camp) in relation to service-provision challenges [34]. Socioeconomic differences between different refugee settings may be partially mitigated by individual financial assistance (distributed by UNHCR and assessed at the country-level). As with other studies that have compared different clinical settings within one national program [35], the clinic setting itself may be the primary consideration. In the present setting, the access that refugees had to HIV services from a leading reference hospital was unusual in comparison to rural, dispersed or camp-based refugee groups. As laboratory monitoring for refugees is implemented according to national protocols, any differences in access to these services among refugees ought to have been similar to routine differences between countries.

Factors identified from these data will help to locate those who might benefit from targeted interventions. To this end, additional counselling for men on HAART, support for those HAART clients who spend lengthy periods in transit to access treatment and care, and those who do not consistently refill their HAART prescriptions as monitored by the pharmacy, could prove to be beneficial. Risk assessments for clients who may travel for extended periods could be implemented to ensure that consistent medication supply is available and contingency plans are in place. Use of mobile phones, either through training in using personal alarms, or more actively through a text-message intervention, may help to address some of these challenges [36, 37]. Given the importance of the pharmacy-based adherence assessment, this measure should be formalised as a routine adherence indicator, linked to medical records, and monitored. When the reported result is poor, this should alert providers and trigger more advanced and expensive testing (e.g. viral loads).

This study had important limitations. Selection bias in the host community group may have affected our findings as response rates were high in both groups, but slightly lower in the host community. Moreover, the recruited host community sample represented 6 % of the target population. As non-participants may have possessed characteristics leading to bias, we compared routine socio-demographic indicators of the study sample with a simple random sample of 150 host community clients drawn from the clinic database. The random sample was statistically similar to the study sample on all socio-demographic indicators with the exception that ethnic Chinese clients were over-represented in the study sample. This could have introduced bias as ethnic Chinese Malaysians tend to have higher household incomes than other ethnic groups in Malaysia [38]. Given that refugees only had routine access to the clinic one day per week, we accounted for the possibility that routine appointments may not have occurred during the study period by making additional efforts (by telephone and/or community representative) to contact refugees who had not been seen in the clinic two weeks prior to the close of recruitment. This procedure facilitated a near-complete sample, while potentially introducing bias linked to these more intensive recruitment efforts that were not similarly implemented among the host community. The cross-sectional design of the study limited our ability to draw any firm causal conclusions, and to accurately measure and classify longer-term viral suppression and adherence [39]. Lastly, as only a single study viral load sample was collected, outcomes may have been subject to sporadic viral escape, or “viral blips” leading to misclassification of the outcome [40–42]. Using ≥500 copies/mL as indicator of viral rebound [43], we compared results in the 40–499 copies/mL range among clients for whom the previous routine viral load was suppressed with those for whom it was not, and found no evidence for differences between groups (Table 2).

This study excluded asylum-seekers who began HAART in their country of origin and who may have been vulnerable to inferior outcomes given the possibility that their HAART was exhausted prior to gaining refugee status and becoming eligible for the national treatment program. These cases were routinely expedited and programs should continue to facilitate and improve pathways to treatment for asylum-seekers. Strengths of the study included detailed adherence assessment using self-report and pharmacy claim measures in accordance with best-practices [33], collection of blood samples using routine phlebotomy, analysis of samples conducted in an independent laboratory on a reliable platform, effective quality control, and the use of well-trained local research staff.

In summary, the high proportion of refugee and host community clients attending this public sector clinic who achieved viral suppression supports the notion that providing HAART to these groups on an equitable basis in this urban setting is both feasible and beneficial. Given the current global reduction of funding for HIV, the future sustainability of HAART for refugees needs to be critically assessed. The Malaysian national program fully subsidises first-line treatments for refugees; however, second-line treatments and virological monitoring are paid for by UNHCR. The concern is that national treatment programs that currently include refugees may opt to exclude them in the future if funding continues to decline. If the goal of universal access to treatment is to be reached and the public health benefits of antiretroviral therapy are to be realised, refugees and other conflict-affected persons must be fully included in country and regional proposals and planning for HIV and AIDS.

## Electronic supplementary material

Below is the link to the electronic supplementary material.

Supplementary material 1 (DOCX 15 kb)
