# Supplementary material for: Is Forced Migration a Barrier to Treatment Success? Similar HIV Treatment Outcomes Among Refugees and a Surrounding Host Community in Kuala Lumpur, Malaysia
Source: AIDS Behav. 2013 Jun 9;18(2):323–34. doi: 10.1007/s10461-013-0494-0 (PMC3905173; doi:10.1007/s10461-013-0494-0)
Supplement: Supplementary file 1 — Supplementary material 1 (DOCX 15 kb) [file 10461_2013_494_MOESM1_ESM.docx]

## Supplementary material

| Table i: Comparison of interviewed and randomly sampled host community clients using data from electronic medical records* | | | |
| --- | --- | --- | --- |
| Factor | Interviewed sample (n_1_) | Random sample (n_2_) | p-value |
| **Female/transgender, n (%)** | 33/144 (23) | 32/150 (21) | 0.74†† |
| **Age in years, median (IQR) (n_1_=148; n_2_=150)** | 42 (34, 49) | 39 (35, 48) | 0.35‡ |
| **Marital status, n (%)** |  |  |  |
| Single | 63/141 (45) | 52/115 (45) | 0.14† |
| Married | 68/141 (48) | 61/115 (53) |  |
| Divorced/widowed | 10/141 ( 7) | 2/115 ( 2) |  |
| **Ethnicity, n (%)** |  |  |  |
| Chinese | 88/145 (61) | 50/140 (36) | <0.001†† |
| Malay | 36/145 (25) | 56/140 (40) |  |
| Tamil/Other | 21/145 (15) | 34/140 (24) |  |
| **Most recent routine viral load, copies/mL (%)** |  |  |  |
| Suppressed <40 | 111/146 (76) | 105/139 (76) | 0.92†† |
| Not suppressed ≥40 | 35/146 (24) | 34/139 (25) |  |
| **Recent routine CD4, median cells/µL (IQR) (n_1_=144; n_2_=149)** | 376 (248, 598) | 350 (202, 486) | 0.07‡ |
| **Time on HAART, median weeks (IQR) (n_1_=140; n_2_=130)** | 184 (59, 324) | 134 (66, 259) | 0.09‡ |
| †Fisher’s exact test ††Chi-squared test ‡Mann-Whitney test | | | |

| Table ii: Unsuppressed viral load by type of adherence measurement, stratified by refugee status (≥25 weeks on treatment) | | | |
| --- | --- | --- | --- |
| Adherence measurement | ≥40 copies/mL, n (%) | Total | p-value† |
| **Host** |  |  |  |
| Dose-by-dose self-report (4 days); n=125 |  |  |  |
| 0+ | 2 (50) | 4 (100) | 0.06 |
| 80+ | 0 ( 0) | 0 ( 0) |  |
| 95+ | 18 (15) | 121 (100) |  |
| Visual analogue scale self-report (1 month); n=125 |  |  |  |
| 0+ | 5 (45) | 11 (100) | 0.002 |
| 80+ | 6 (22) | 27 (100) |  |
| 95+ | 9 (10) | 87 (100) |  |
| Pharmacy claim adherence (24 months); n=121 |  |  |  |
| 0+ | 4 (31) | 13 (100) | 0.10 |
| 80+ | 5 (16) | 32 (100) |  |
| 95+ | 9 (12) | 76 (100) |  |
| **Refugee** |  |  |  |
| Dose-by-dose self-report (4 days); n=152 |  |  |  |
| 0+ | 3 ( 50) | 6 (100) | 0.07 |
| 80+ | 0 ( 0) | 1 (100) |  |
| 95+ | 20 ( 17) | 114 (100) |  |
| Visual analogue scale self-report (1 month); n=121 |  |  |  |
| 0+ | 3 (43) | 7 (100) | 0.06 |
| 80+ | 6 (25) | 24 (100) |  |
| 95+ | 14 (16) | 90 (100) |  |
| Pharmacy claim adherence (24 months); n=113 |  |  |  |
| 0+ | 4 (44) | 9 (100) | 0.004 |
| 80+ | 8 (32) | 25 (100) |  |
| 95+ | 10 (13) | 79 (100) |  |
| †Chi-squared test for trend (Cochran-Armitage test) |  |  |  |
